# Development and Clinical Validation of RT-LAMP-Based Lateral-Flow Devices and Electrochemical Sensor for Detecting Multigene Targets in SARS-CoV-2

**DOI:** 10.3390/ijms232113105

**Published:** 2022-10-28

**Authors:** Apoorva Saxena, Pawankumar Rai, Srishti Mehrotra, Samiya Baby, Suman Singh, Vikas Srivastava, Smriti Priya, Sandeep K. Sharma

**Affiliations:** 1 Food, Drug & Chemical Toxicology Group, CSIR-Indian Institute of Toxicology Research, Vishvigyan Bhawan, 31, Mahatma Gandhi Marg, Lucknow 226001, India; 2 Academy of Scientific and Innovative Research (AcSIR), Ghaziabad 201002, India; 3 System Toxicology & Health Risk Assessment Group, CSIR-Indian Institute of Toxicology Research, Vishvigyan Bhawan, 31, Mahatma Gandhi Marg, Lucknow 226001, India; 4 Agrionics Post Harvest Material Science and Sensor Applications, CSIR-Central Scientific Instruments Organization, Chandigarh 160030, India

**Keywords:** SARS-CoV-2, COVID-19, RT-LAMP, multi-targets, lateral flow devices, electrochemical sensor

## Abstract

Consistently emerging variants and the life-threatening consequences of SARS-CoV-2 have prompted worldwide concern about human health, necessitating rapid and accurate point-of-care diagnostics to limit the spread of COVID-19. Still, However, the availability of such diagnostics for COVID-19 remains a major rate-limiting factor in containing the outbreaks. Apart from the conventional reverse transcription polymerase chain reaction, loop-mediated isothermal amplification-based (LAMP) assays have emerged as rapid and efficient systems to detect COVID-19. The present study aims to develop RT-LAMP-based assay system for detecting multiple targets in N, ORF1ab, E, and S genes of the SARS-CoV-2 genome, where the end-products were quantified using spectrophotometry, paper-based lateral-flow devices, and electrochemical sensors. The spectrophotometric method shows a LOD of 10 agµL^−1^ for N, ORF1ab, E genes and 100 agµL^−1^ for S gene in SARS-CoV-2. The developed lateral-flow devices showed an LOD of 10 agµL^−1^ for all four gene targets in SARS-CoV-2. An electrochemical sensor developed for N-gene showed an LOD and E-strip sensitivity of log 1.79 ± 0.427 pgµL^−1^ and log 0.067 µA/pg µL^−1^/mm^2^, respectively. The developed assay systems were validated with the clinical samples from COVID-19 outbreaks in 2020 and 2021. This multigene target approach can effectively detect emerging COVID-19 variants using combination of various analytical techniques at testing facilities and in point-of-care settings.

## 1. Introduction

SARS-CoV-2 is a highly transmissible and pathogenic variant of coronavirus that was first reported in late 2019 and since then, it has been associated with severe respiratory sickness [1]. The World Health Organization designated the virus outbreak as the COVID-19 pandemic owing to the rapid rate of its transmission, which posed a major threat to public safety, global economies, and general wellbeing [2]. Every part of the world has been significantly impacted by the devastating COVID-19 pandemic, which has resulted in over 300 million confirmed cases and over 5 million fatalities [3]. The exponential rise of COVID-19 patients and emerging variants have prompted healthcare professionals to focus on developing specialized and sensitive diagnostic tools [4]. Three types of detection systems are currently in use for the diagnosis of COVID-19: PCR-based amplification and detection, CRISPR-based detection, and protein-based detection systems [5]. The detection of SARS-COV-2 viral RNA is confirmed by Nucleic Acid Amplification Tests (NAAT) [6]. For the detection of viral RNA such as SARS-CoV-2, RT-PCR is the most sensitive method, where the total RNA of the virus and host is reverse transcribed by reverse transcriptase into complementary DNA (cDNA). The RT-PCR is then used to quantitively amplify the target of interest from cDNA [7,8,9]. RT-PCR requires specialized instruments, expertise, and time; hence, it cannot be used in point-of-care settings [10]. Several antibody-based detection techniques coupled with paper-based lateral-flow strips have been reported for the fast detection of COVID-19; however, these technologies detect IgG and IgM antibodies against SARS-CoV-2, that offers information on the exposure history of patients [11,12]. Antigen-based immunological assays are quick but are less sensitive [13]. For the detection of mutations in the viral genome, sequencing-based molecular approaches such as next-generation sequencing are used; however, this approach is unsuitable for routine diagnosis of SARS-CoV-2 [14]. The cDNA produced from SARS-CoV-2 RNA is hybridized with appropriate probes using a microarray-based nucleic-acid hybridization approach. This approach, on the other hand, may not be able to detect scarce viral genes in small samples [15]. Simple isothermal-based molecular detection techniques such as Loop-Mediated Isothermal Amplification (LAMP) and Recombinase Polymerase Amplification (RPA) have been widely employed to develop quick detection methods for COVID-19 [16]. Although an integrated suit-case-based RT-RPA was created for the detection of three SARS-CoV-2 gene targets (RdRP, E, and N), the clinical sensitivity of the assay and specificity were not investigated [17]. The SHERLOCK assay employed RPA as a pre-amplification technology and a Cas9-based strategy for the detection of COVID-19. The test is difficult due to the simultaneous use of RPA and CRISPR-Cas in a single detection apparatus [18].

LAMP targets specific sequences with up to six different sets of primers. As reverse transcriptase enzyme is included in the LAMP reaction, the assay may detect viral RNA in a single tube [19]. As compared to PCR, the LAMP test has a higher affinity for surviving the inhibitory effect of irrelevant nucleic acids prevalent in clinical samples, making it a superior option for usage in clinical settings [20]. The LAMP reaction has a high amplification efficiency and can be used with fluorescent dyes to visually determine the end products [21]. One-step RT-LAMP assay targeting the two genes ORF1a and N-gene has been reported with an accuracy rate of 95% [22]. An electrochemical sensor based on a rolling circle mechanism, an isothermal amplification technique, has also been reported for the detection of S-gene and N-gene of SARS-CoV-2 [23].

Most real-time PCR assays target at least two genes to avoid cross-reactivity with other endemic coronaviruses [24]. None of the single molecular targets could alone potentially detect SARS-CoV-2 [25]. However, the genome of SARS-CoV-2, like other viruses, is highly susceptible to mutations that could cause genetic drift, necessitating the use of a multi-target approach for specific detection of the virus [26]. To detect emerging mutants and address point-of-care applications, RT-LAMP was used, and the amplification products were quantified using absorbance, fluorescence, lateral flow, and electrochemical assays for N-gene, ORF1ab-gene, E-gene, and S-gene in SARS-CoV-2. The dual-labeled amplified products were observed on lateral flow paper-based strips for the detection of SARS-CoV-2 viral mRNA. The spectrophotometric and ready-to-use lateral flow strips were validated using clinical samples for all four gene targets.

## 2. Results

### 2.1. Study Design for RT-LAMP-Based Lateral Flow Device and Electrochemical Sensors for SARS-CoV-2 Detection

SARS-CoV-2 target regions were selected from the National Centre for Biotechnology Information (NCBI) database. The DNA oligos of N-gene, ORF1ab-gene, E-gene, and S-gene of the SARS-CoV-2 genome were customized (Supplementary Figure S1). To optimize the LAMP assay conditions, the customized DNA fragments/oligos were used as templates. The primers were selected on the basis of low primer artifacts, clear negative controls, and better amplification in the positive control for RT-LAMP assay. The primers were labeled to develop lateral-flow devices using the Milenia HybriDetect strips for N-gene, ORF1ab-gene, E-gene, and S-gene and in-house nucleic-acid-based lateral-flow devices for N-gene of SARS-CoV-2. Further, an electrochemical sensor targeting the N-gene based on the probe-amplicon interaction was developed and validated.

### 2.2. PCR Amplification of Customized SARS-CoV-2 DNA Sequences

SARS-CoV-2 customized DNA oligos were amplified using specific primers (Supplementary Table S4). Following PCR amplification of customized DNA sequences, an amplicon size of 1129 bp (N-gene), 855 bp (ORF1ab-gene), 210 bp (E-gene), and 245 bp (S-gene) was observed on agarose gel (Supplementary Figure S2). The main stocks of all purified customized DNA were diluted to 100 ngµL^−1^ in TE buffer and serially diluted 10-fold for further use.

### 2.3. Development of LAMP Assay for Customized DNA Sequences

To carry out the SARS-CoV-2 DNA-based LAMP assay, sixteen sets of primers from the literature (Supplementary Table S1) and four self-designed sets of primers were used. LAMP reactions were standardized for 60 min at 63 °C using a positive control (DNA) and no template control (NTC). Seventeen (17) of the total primer sets at concentrations of 0.2 M (F3 and B3), 0.8 M (LF and LB), and 1.6 M (FIP and BIP) were found optimum (Supplementary Table S1).

### 2.4. In Vitro Transcription of mRNA and RT-LAMP Assay Using FAM and Biotin Labelled Primers

**Figure 1:**
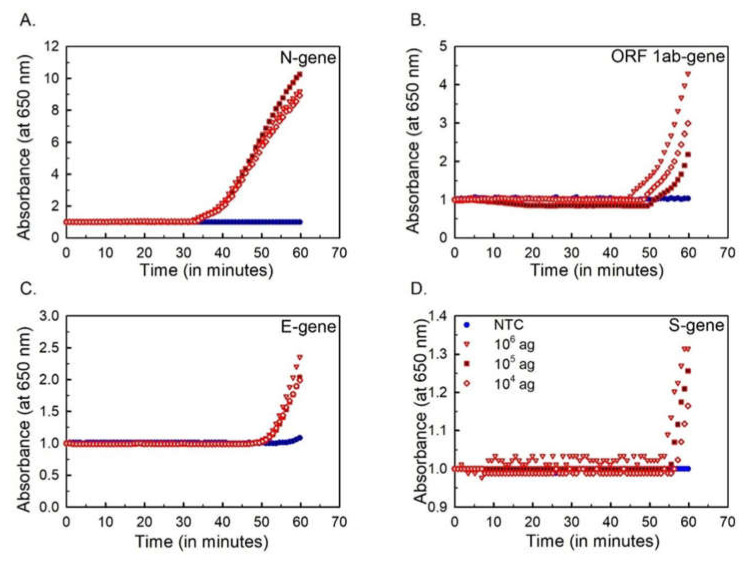
**Sensitivity of RT-LAMP assay.** Amplification curves for in vitro transcribed RNA (10^6^ agµL^−1^, 10^5^ agµL^−1^, and 10^4^ agµL^−1^) between the absorbance at wavelength 650 nm and time of amplification.

In vitro transcription of customized DNA fragments was performed to obtain the viral mRNA segments for RT-LAMP assay. The purity and concentration of the in vitro transcribed mRNA were optimized to carry out the RT-LAMP experiments (Supplementary Table S3). For RT-LAMP amplification, the selected primer sets (7; Supplementary Table S1e) were tagged at 5′ end with 6-FAM and biotin. The RT-LAMP assay with tagged primer sets (N-5, N-9, ORF1ab-1, ORF1ab-2, E-1, S-1, and S-4) was performed by incorporating the labeled primers with unlabeled primer sets under the same conditions as in LAMP assay. Four sets of primers for N-9, ORF1ab-2, E-1, and S-4 were finally selected. The sensitivity of the RT-LAMP assay was assessed with 10-fold serial dilutions of standard in vitro transcribed RNA from 10^6^ agµL^−1^ to 1 agµL^−1^. The absorbance at 650 nm for every 1 min time interval at 63 °C was recorded for each target gene. RT-LAMP assay could detect 10 agµL^−1^ of mRNA viral standard for N-gene, ORF1ab-gene, and E-gene, whereas for S-gene sensitivity was 100 agµL^−1^ (Figure 1). The RT-LAMP assay with tagged primers was repeated three times to determine the reproducibility of the assay.

### 2.5. RT-LAMP LFD-Based Detection of SARS-CoV-2 Using In Vitro Transcribed Viral mRNA

**Figure 2:**
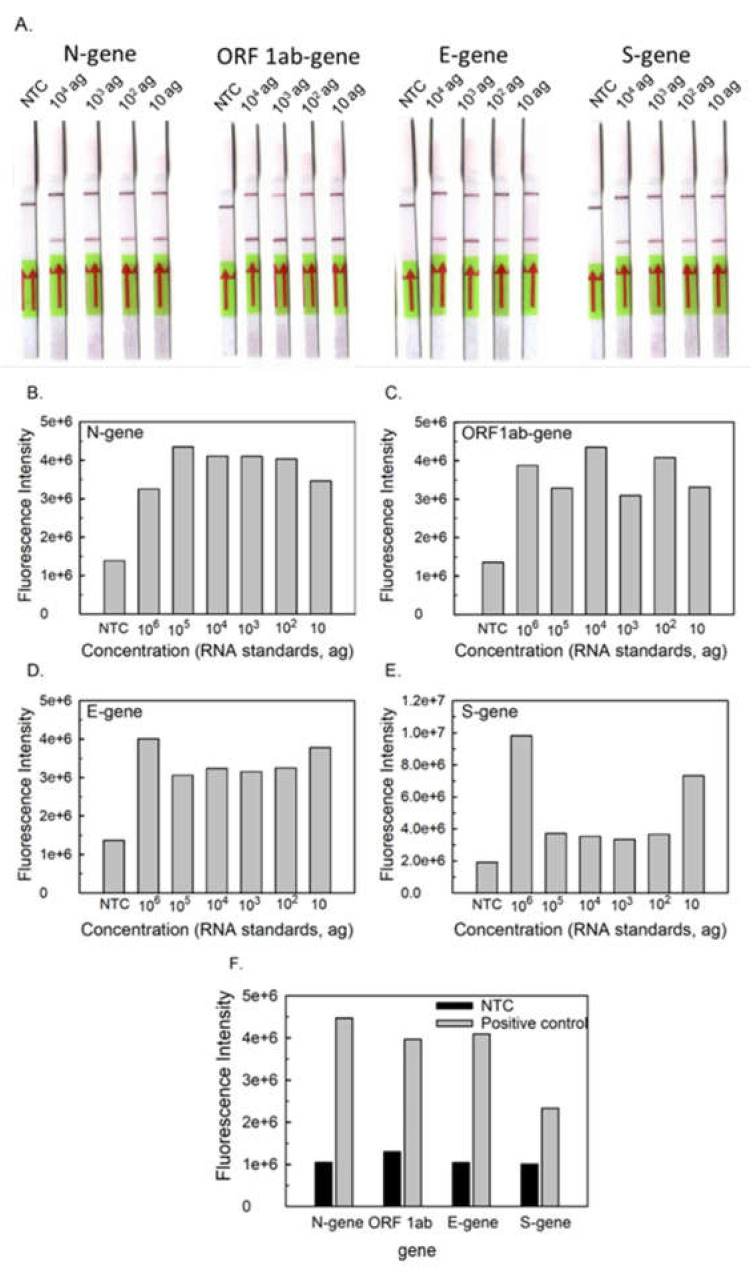
**Sensitivity of RT-LAMP-based LFD of SARS-CoV-2 using in vitro transcribed viral mRNA.** (**A**) LFD of RT-LAMP amplified in vitro transcribed standard RNA and no template control (NTC); (**B**–**E**) Fluorescence detection of 10-fold diluted RT-LAMP amplified products of in vitro transcribed mRNA standards using SYBR Green I dye. (**F**) Fluorescence detection of RT-LAMP amplified products using SYBR Green I dye for no template control (NTC) and positive control (PC; 1.0 ngµL^−1^).

RT-LAMP-LFD assay was performed with 1 ngµL^−1^ of each target mRNA of N-gene, ORF1ab-gene, E-gene, and S-gene. The reaction was performed with the tagged and non-tagged primer sets (N-9, ORF1ab-2, E-1, and S-4). All the targets after amplification provide a test and control line in positive template control, while the negative template control shows only a control line at 100-fold dilution of amplified products. The lateral-flow device based on RT-LAMP amplification could detect 10 agµL^−1^ of standard in vitro transcribed RNA of N-gene, ORF1ab-gene, E-gene, and S-gene (Figure 2A). The fluorescence intensity of the amplified samples is depicted in Figure 2B–F.

### 2.6. RT-LAMP-LFD-Based Detection of SARS-CoV-2 Viral mRNA in Clinical Samples

**Figure 3:**
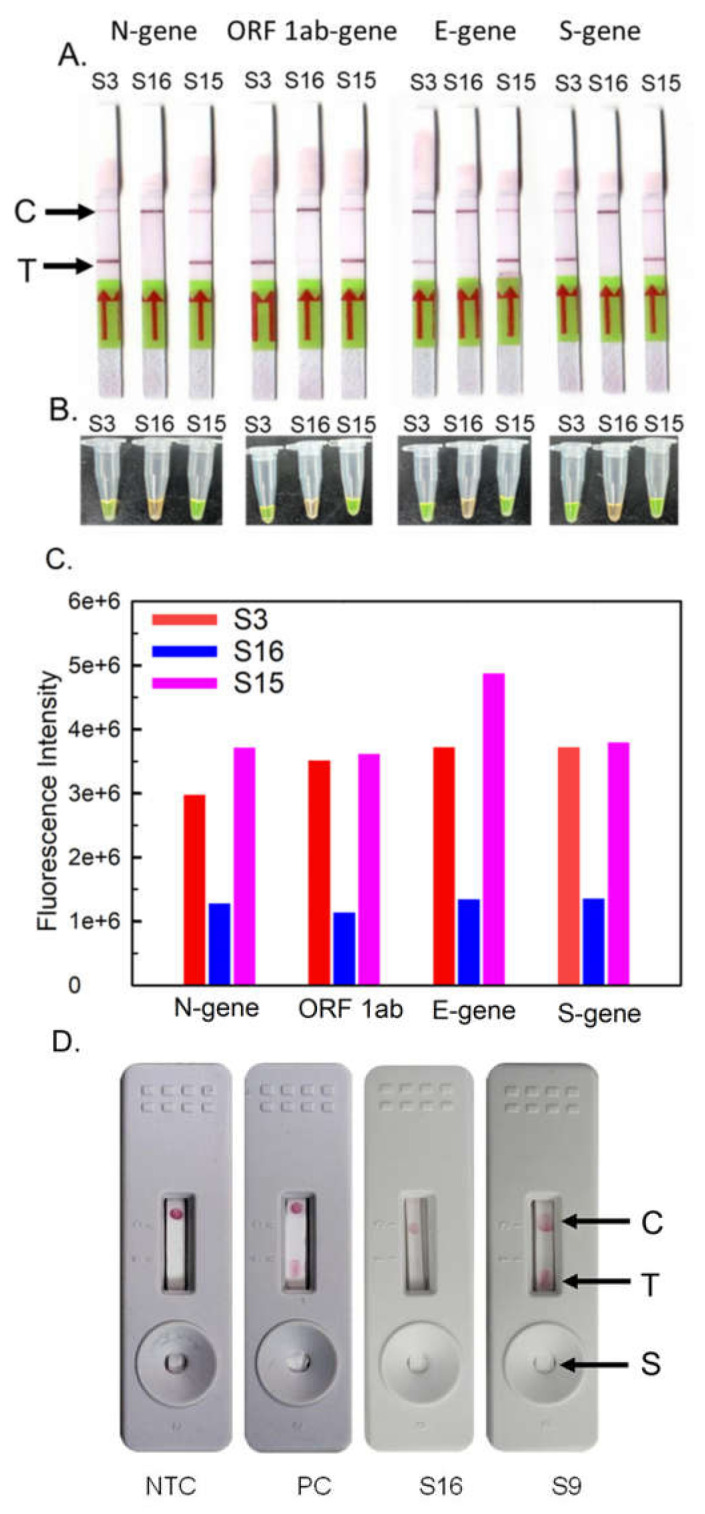
**LFDs and visual detection for RT-LAMP amplified SARS-CoV-2 viral mRNA from patient’s oropharyngeal and nasopharyngeal swab samples.** (**A**) Lateral flow devices for three clinical samples S3 (Ct value: 12.978), S16 (Ct value: 38.539), and S15 (Ct value: 27.99). (**B**) Visual detection of RT-LAMP amplified clinical samples using SYBR Green I dye. (**C**) Fluorescence detection of RT-LAMP amplified clinical samples after 10-fold dilution using SYBR Green I dye. (**D**) Nucleic-acid-based lateral flow device (NALFD) for the RT-LAMP amplified samples; From left, cassette 1: Negative template control and cassette 2: customized N-gene (10 ng µL^−1^) as positive control; cassette 3: RT-LAMP amplified SARS-CoV-2 clinical mRNA targeting N-gene; Negative sample S16 (real-time PCR Ct value 38.539) and cassette 4: Positive sample S9 (real-time PCR Ct value 20.149).

One primer set for each target region of SARS-CoV-2 was selected for RT-LAMP-LFD assay. The separate reactions of RT-LAMP assay for four targets of SARS-CoV-2 (N-9, ORF1ab-gene-2, E-1, and S-4) were performed using 16 samples. A test and control line were observed in positive samples, while negative samples showed only the control line on Milenia HybriDetect dip strips for all four targets of SARS-CoV-2. The results are shown in Table 1 and Figure 3A. The results obtained from the RT-LAMP-LFD were in correlation with Ct-values observed by RT-PCR except for the samples S11 and S12, which tested negative with ORF1ab-gene and E-gene and samples S4, S5, S8, S9, and S10 tested negative for S-gene. The amplified products of three clinical samples, S3, S16, and S15, of Ct values 12.978, 38.539, and 27.99 were also visualized using SYBR Green I dye, and fluorescence was recorded (Figure 3B). The difference in the fluorescence intensity depicted in the form of bar graphs clearly indicates the negative and positive samples (Figure 3C).

The lab-made strips were also evaluated with two clinical samples for proof of concept. Red spots were observed in the sample having a Ct value of 20.149 at the test and control line, while the sample having a Ct value of 38.539 shows a single red spot at the control line. The results obtained from the lab-made strips were in concordance with the real-time PCR assay results, which specified the Ct value 38.539 as a negative sample and the Ct value 20.149 as a positive sample. Clear distinction regarding the presence of a single red spot at the control line and two red spots at the test and control line indicates the negative template control and standard positive control, respectively (Figure 3D).

### 2.7. Surface Characterization of Screen-Printed Gold Electrode

**Figure 4:**
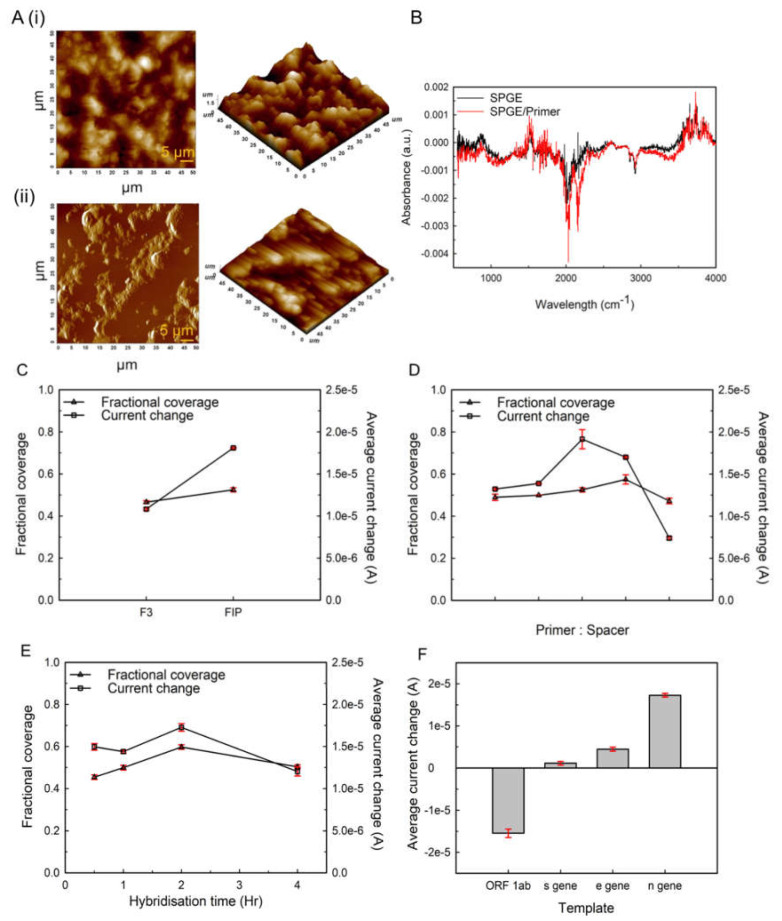
**Surface characterization of SPGE-based electrochemical sensor for SARS-CoV-2;** (**A**) Topographical scan in height mode at a scale of 5 µm of (**i**) unmodified, (**ii**) modified SPGE/β-ME/ssDNA probe. (**B**) FTIR spectra of unmodified and modified SPGE/β-ME/ssDNA probe. (**C**) Selection of appropriate primer (F3 and FIP) for target gene (N-gene). (**D**) Optimization of ratio of primer to spacer for development of Self-assembled Monolayer (SAM). (**E**) Selection of optimum time-point for hybridization of primer with target gene. (**F**) Establishment of response against non-specific targets.

Figure 4A shows surface topography (height) of bare screen-printed Au electrode (unmodified SPGE) (Figure 4Ai), and β-ME/ssDNA probe/screen printed Au electrode (Figure 4Aii) (modified SPGE) obtained using AFM (scale = 5 µm). Figure 4(Ai,Aii) shows uniform distribution of rough granular structure, whereas, after immobilization of probe and spacer onto the modified SPGE, the roughness increased from a spacer-induced alignment of ssDNA monolayer onto modified SPGE that filled the pores present on the Au surface. Additionally, the phase images (Supplementary Figure S3) validate the height images, showing variation in phases after immobilization of the β-ME/ssDNA probe onto the SPGE surface as compared to the unmodified SPGE [27,28,29].

The FTIR spectra of the modified and unmodified screen-printed gold electrode were obtained in absorbance mode, as shown in Figure 4B. It is evident from the spectra that modified SPGE has vibrational bands and functional group for ssDNA and β-ME. Peaks between 1310–1870 cm^−1^ and 901.86 cm^−1^ are linked to N-H bending and stretching of DNA bases purine and pyrimidine ring [29,30]. The modified electrode has a characteristic -SH band between 2450 and 2650 cm^−1^ due to the rupture of the SH bond and formation of the S-Au bond; the strength of the peak in the modified electrode increases with peak broadening between 1950 and 2250 cm^−1^ [31]. Peak suppression at 2525.09 cm^−1^ and 2634.39 cm^−1^ also confirms the S-H bond cleavage indicating uniform assembling of β-ME/ssDNA probe on the Au electrode. Additionally, -CH_2_- bands appeared at 2807.25 cm^−1^, 2987.36 cm^−1^, and 3010.04 cm^−1^, clearly demonstrating the strong S-Au bond; other groups in the β-ME molecule have no effect on its assembly [29,31,32].

### 2.8. Electrochemical Sensor for Detection of SARS-CoV-2

Initial experiments were performed to determine the primer type that binds strongly to the template. Two primers (N-9) targeting N-gene used in RT-LAMP assay, namely, F3 and FIP, were used for this purpose. The primers (0.5 µM) were added to the spacer (0.5 µM): beta-mercaptoethanol (β-ME) and immobilized on the gold electrode. The immobilization was undertaken overnight (~20 h) at 37 ℃, and the electrode was washed before adding the complementary DNA (cDNA). The hybridization of cDNA was conducted for 2 h at 55 ℃. The differential pulse voltammogram (Supplementary Figure S4a,c,e,g) was used to calculate average current change (ΔI), and the Rct value from the Nyquist plot (Supplementary Figure S4b,d,f,h) was used to calculate fractional coverage (Ψ_FC_). Figure 4C shows the average current change and the corresponding fractional coverage of the gold electrode using the two primers. A higher degree of change in current was produced by FIP due to its larger size (41 bp), because of which it is able to bind to the cDNA strongly. F3 is comparatively smaller in size (18 bp), which affects its accessibility for hybridization with cDNA and, subsequently, the electron-transfer rate. FIP was used for all the further experiments.

Subsequently, the assembly of the spacer and primer (FIP) on the electrode surface was optimized. FIP primer and β-ME were immobilized on the electrode surface in the ratio 1:0, 1:0.5, 1:1, 0.5:1, and 0:1. The modified electrodes were incubated with cDNA at 55 °C to facilitate hybridization. After 2 h of incubation, DPV and EIS of the modified electrode were recorded, and Ψ_FC_ and ΔI were determined. Maximum Ψ_FC_ of 0.525 ± 0.01 and ΔI of 1.91 × 10^−5^ ± 1.14 × 10^−6^ were observed for FIP:β-ME concentration 1:1 (Figure 4D).

The addition of primer or the spacer on the electrode surface induced resistance to current flow, thereby resulting in a decreased peak current (I_p_). The magnitude of reduction is dependent on the quantity and orientation of the primer and spacer. In the absence of a spacer, the primer forms a misaligned monolayer that increases the steric hindrances and reduces the availability of the primer for hybridization (Supplementary Figure S7a). At 1:0 (FIP:β-ME) concentration, the length of the monolayer is short and conducive for a high electron transfer rate resulting in increased I_p_ and a low ΔI (Supplementary Figure S4a). On increasing the concentration of the spacer, the non-specific interaction between the primer and gold surface decreases, and the compactness on the electrode surface improves. The non-specific bonds are replaced with strong Au–S (thiol group in β-ME) bond. As a result, primers arrange themselves in a rigid, upright position, thus improving their accessibility. The increased length of SAM hinders the electron transfer resulting in a decrease in I_p_. At FIP:β-ME concentration, 1:0.5, 1:1, 0.5:1, the fraction coverage of the electrode is also higher than that on FIP:β-ME concentration, 1:0, and 0:1. From here, the concentration of FIP:β-M, 1:1 was determined to be conducive to higher primer accessibility for hybridization.

The effect of hybridization time on the activity of the modified electrode (1:1) was determined by varying time from 30 min to 4 h at 55 °C. DPV was performed, and ΔI was calculated. The maximum change in I_p_ was observed for 2 h of hybridization time (Figure 4E). The current change decreases considerably as the hybridization time is increased to 4 h. ΔI for a hybridization time of 30 min and 1 h was also less than ΔI for 2 h.

The response of non-complementary amplified products (Nc products), namely, ORF1ab-gene, E gene, and S gene against the modified electrode (1:1), after 2 h of hybridization was established. A significant decrease in ΔI was observed for Nc Products (Figure 4F). The current change brought about by the addition of non-complementary amplified products suggests their non-specific binding with the primer due to H-bond interaction. These additional non-specific bonds on the surface of the modified electrode capture the active species closer to the electrode surface, facilitating electron transport. Hence, it could be inferred that ΔI corresponding to non-complementary amplified products is an indicator of additional elements of non-specific binding and not hybridization. Analyses of the template concentration ranging from 100 fgµL^−1^ to 1 ngµL^−1^ showed a linear dependence between log ΔI and log template with linear regression equation y = 0.6453x − 6.077 and R^2^ = 0.91 following first order. LOD was calculated to be log 1.79 ± 0.427 pgµL^−1^. The sensitivity of the E-strip, calculated by the formula: Slope of calibration plot (log µA/pg µL^−1^)/Active Surface Area (mm^2^) was log 0.067 µA/pg µL^−1^/mm^2^ (Supplementary Figure S4b,c).

The response of the sensing electrode was verified against RT-LAMP amplified viral mRNA isolated from the nasopharyngeal and oropharyngeal swab samples of SARS-CoV-2. The results, along with the corresponding Ct value and the inference of the same, are mentioned in Table 2.

## 3. Discussion

Since the emergence of the COVID-19 pandemic, a crucial factor in its management is the availability of rapid and reliable diagnostic methods. Several onsite detection kits are available; still, developing countries with limited infrastructure are struggling with delayed detection of infections. The early and specific detection of SARS-CoV-2 can effectively prevent community transmission and risk management of COVID-19. Real-time RT-PCR is a reliable and primary choice for the routine detection of COVID-19, but it requires sophisticated equipment and technical expertise to interpret the results. Additionally, owing to a limited choice of probe sequence and target gene regions of SARS-CoV-2, which may be susceptible to mutations, this raises concerns about false detections [10]. Another popular method is the antibody-based immunological tests, which are used as a secondary tool for community screening; however, these methods determine the active humoral immunity and not the active viral load [5,33]. The paper-based or chip-based electrochemical sensors using antisense oligonucleotide markers for specific genes detection of SARS-CoV-2 are available but are limited in use [34]. Isothermal nucleic acid-based amplification techniques appear to be a better alternative for the diagnosis of the disease in resource-limited laboratories [35]. RT-LAMP has been previously established for the detection of various viral diseases such as SARS coronaviruses, MERS-CoV, Avian influenza viruses, Ebola viruses, etc. [36,37]. The recently developed methods and kits for the detection of SARS-CoV-2 RNA, such as the SHERLOCK diagnostic test [38], DETECTR [39], and FELUDA [40], are based on isothermal nucleic acid amplification assays and CRISPR-Cas systems. These detection methods are based on single conserved gene targets of SARS-CoV-2; however, the rapid evolution and genetic diversity in SARS-CoV-2 may lead to false negatives and reduced test efficiencies [40]. The high mutation rates, deletion, and insertions resulted in the emergence of new variants of concern may be detected if multiple genome regions are targeted for detection [41].

In the present study, four different gene regions (N, ORF-1ab, E, and S) of SARS-CoV-2 have been targeted. The dual-labeled amplified RT-LAMP products were analyzed using different methods such as spectrophotometry, paper-based lateral-flow devices, and electrochemical sensors.

The end products of the RT-LAMP assay were analyzed by absorbance and fluorescence spectrophotometry. The change in turbidity due to the accumulation of magnesium pyrophosphate ions was recorded in real-time at a wavelength of 650 nm for one hour at 63 °C. The pyrophosphate ions are accumulated in the reaction during amplification due to the conversion of deoxyribonucleotide triphosphates to deoxyribonucleotide monophosphates and pyrophosphate ions. These pyrophosphate ions combine with magnesium ions to form magnesium pyrophosphate [42]. The threshold values for positive samples were taken as >0.1 OD on the basis of maximum OD of 0.1 attained by the amplification of negative samples in 60 min. The limit of detection of absorbance-based detection method was found to be 10 agµL^−1^ for the N-gene (corresponds to 1.61 × 10^2^ copies of customized DNA fragment), ORF1ab-gene (2.13 × 10^2^ copies of customized DNA fragment) and E-gene (8.69 × 10^2^ copies of customized DNA fragment), and 100 agµL^−1^ (7.44 × 10^3^ copies of customized DNA fragment) for the S-gene.

Similarly, fluorescence spectrophotometry of the RT-LAMP amplified products with SYBR Green I dye was measured at the excitation wavelength of 498 nm and emission wavelength of 522 nm. The limit of detection for all target genes (N-gene, ORF1ab-gene, E-gene, and S-gene) was found to be 10 agµL^−1^, as real-time detection by fluorescence is a sensitive method with a threshold value of 1.3 × 10^6^ based on the maximum fluorescence intensity attained by the negative samples in 60 min. The threshold value of samples >1.3 × 10^6^ were regarded as positive samples. The absorbance and fluorescence of RT-LAMP amplified products is a simple detection method for multi-target genes of SARS-CoV-2 and provide an immediate and quantitative response.

Further, the dual-labeled RT-LAMP products were analyzed by ready-to-use Milenia HybriDetect lateral flow dip strips to detect the amplified products tagged with FAM and biotin. These strips contain FITC/FAM ligand conjugated with gold nanoparticles at the conjugation pad and biotin ligand at the test line. The control line has a species-specific antibody. All the positive samples contain both the test and control bands, whereas negative samples contain only the control band. The limit of detection of LFDs was 10 agµL^−1^ for each gene target (Figure 2A). These strips have the potential for multiplexing in future applications [43].

As a proof of concept, an in-house lateral flow device was also developed for the detection of N-gene of SARS-CoV-2 using dual-labeled RT-LAMP amplicons. The developed prototype was modified for the double run method [29], where only 1.0 µL of the conjugated gold nanoparticles were required for the screening of positive and negative COVID-19 samples.

Further, an electrochemical sensor based on a screen-printed gold electrode for accurate detection of N-gene in SARS-CoV-2 was developed. The specific LAMP primer (N9; Supplementary Table S1e), based on its amplification efficiency, was selected along with an organic spacer, β-ME, to aid primer orientation resulting in the compact binding surface idyllic for electrochemical biosensor. A significant increase in the current change with FIP primer and spacer (0.5 µM) was observed, suggesting suitable size and orientation of the primer accessibility for hybridization and the electron exchange potential of the sensing surface. The N-gene amplified by RT-LAMP end product was allowed to hybridize with primer-immobilized SPGE for 1 h, 2 h, 3 h, and 4 h. Two hours of hybridization was found to be optimum for the electrochemical signal generation (Figure 4A). The electrochemical sensor with FIP primer for non-complementary targets showed a non-significant change in peak current, making the method selective towards N-gene (Figure 4B). The limit of detection (LOD) was found to be log 1.79 ± 0.427 pg µL^−1^, and the sensitivity of the E-strip was log 0.067 A/pg µL^−1^/mm^2^.

The clinical validation of the lateral flow dip strips and the electrochemical sensor was conducted with the RNA samples of patients (Table 1 and Table 2). LFDs were validated with 16 RNA samples, where false negative results were observed with two samples (S11 and S12) for ORF1ab-gene and E-gene targets. However, due to the multigene strategy, samples tested positive with the N-gene and S-gene, thus eliminating the chances of false negative detections (Table 1). Among the clinical samples, assorted samples from the outbreaks in 2020 and 2021 were also tested. For the samples from 2021, all five RNA samples (S4, S5, S8, S9, and S10) tested negative for S-gene but showed positive results for all other target regions. This implies the importance of a multi-target approach for the established assays. The results of the LFD-based RT-LAMP assay were found to be in concordance with the RT-PCR results, implying the sensitivity, specificity, and potential of the developed assays for the diagnosis of COVID-19. The electrochemical sensor for N-gene was validated with 15 RNA samples from COVID-19 patients. The results were in concordance with RT-PCR results, except for the sample having a Ct value of 16.205, which was found to be false negative with the electrochemical sensor.

Most of the available RT-LAMP-based molecular diagnostic kits utilize a single or two-target approach. With the emergence of different variants, such as the recent variant of concern, Omicron (B.1.1.529), with more than 50 mutations in spike proteins and receptor binding domain (RBD) [44], a multi-target approach is essential. Our study used a combination of detection systems that allowed us to confirm SARS-CoV-2 and variants precisely in the infected individuals and eliminated chances of missing virus detection due to mutations.

## 4. Materials and Methods

### 4.1. Designing of Primers for LAMP Assay and In Vitro Transcription of Viral mRNA

From the previously published literature, sixteen specific primers targeting the N-gene, ORF1a-gene, E-gene, and S-gene were chosen for the LAMP assay. LAMP primer designing software was used to create four new primers (PrimerExplorerV5). The desalted LAMP primers (25 nmole) were synthesized by Integrated DNA Technologies (IDT, USA) and reconstituted in Tris-EDTA buffer (pH 7.4) (Supplementary Table S1a–d). LAMP primers (25 nmole, desalted) were selected and labeled at the 5′ end with 6-FAM and biotin for the establishment of the lateral-flow assay (Supplementary Table S1e).

Primers targeting the N-gene (1129 bp), ORF1ab-gene (855 bp), E-gene (210 bp), and S-gene (245 bp) were designed for the artificial synthesis of positive control viral mRNA (IDT, Coralville, IA, USA). For in vitro transcription of viral mRNA, forward primers for all targets contain a T7 RNA polymerase promoter (Supplementary Table S2).

### 4.2. Preparation of Custom DNA Oligos and PCR Amplification

The control template RNA for the LAMP assay was generated by the artificial synthesis of DNA oligos (IDT, Coralville, IA, USA) from known SARS-CoV-2 sequences obtained from the Global Initiative for Sharing All Influenza Data (GISAID) and NCBI databases. The N-gene, ORF1ab-gene, E-gene, and S-gene sequences were obtained from the NCBI database using the complete genome of SARS-CoV-2 isolate Wuhan-Hu-1 (NCBI Reference sequence: NC 045512.2). Supplementary Figure S1 shows the position and sequences of the customized DNA. Phusion™ High-Fidelity PCR kit (#F553L, ThermoFisher Scientific, Waltham, MA, USA) was used to amplify SARS-CoV-2 specific artificially synthesized DNA fragments. In brief, customized DNA fragments for the N-gene and ORF1ab-gene (150 ng each), E-gene, and S-gene (50 ng each) were amplified with forward and reverse primers (1 pmoleµL^−1^) and Phusion™ DNA polymerase (0.02 UµL^−1^). The initial denaturation was carried out for 30 s at 98 °C. After that, 35 cycles of denaturation at 98 °C for 10 s, annealing at 55 °C for 30 s, and extension at 72 °C for 30 s were performed. The final extension lasted 10 min at 72 °C. For all four targets, the PCR cycling parameters remained constant (N-gene, ORF1ab-gene, E-gene, and S-gene). FQIAquick^®^ Gel Extraction Kit (#28704, QIAGEN, Hilden, Germany) and QIAquick^®^ PCR and Gel Cleanup Kit (#28104, QIAGEN, Hilden, Germany) were used to purifying the amplified products. On the 1.5 percent agarose gel electrophoresis, the expected bands were visible. The purified DNA was further used as a template for the development of LAMP assay and in vitro synthesis of viral mRNA.

### 4.3. LAMP Reaction Using Artificially Synthesized SARS-CoV-2 Viral DNA Fragment

For LAMP-based amplification of SARS-CoV-2 DNA, 19 sets of primers (10 for N-gene, 4 for ORF1ab-gene, 1 for E-gene, and 4 for S-gene) were initially chosen. The screening of primer sets was based on clear negative control, fewer primer artifacts, and significant positive control amplification (Supplementary Table S1). The primer concentrations and reaction conditions of the selected primer sets were further optimized. The isothermal amplification was performed in a 25 µL reaction containing 10× of isothermal buffer, 6 mM of MgSO_4_, 1.4 mM of dNTPs, 800 mM of betaine, 8 U of Bst 2.0 WarmStart DNA polymerase (#M0538, New England Biolabs, Ipswich, MA, USA), 1.6 M of forward inner primer (FIP) and backward inner primer (BIP), 0.8 M of loop forward (LF) and loop backward (LB), 0.2 µM of outer forward (F3) and outer backward (B3) primer, 1 µL of standard customized DNA oligo as positive control template (1 µL) and nuclease-free water (#AM9937, Thermo Fisher Scientific, Waltham, MA, USA) at 63 °C for 1 h. Results were monitored by absorbance at 650 nm for 60 min at every 1-min interval in Cytation 5 Imaging reader, Biotek, Winooski, VT, USA. Amplified products were further visualized in visible light by the addition of 1 µL of 1000× SYBR Green I, and fluorescence readings were observed at 498 nm of excitation wavelength and 522 nm of emission wavelength in Cytation5 Imaging reader, Biotek, Winooski, VT, USA. Further, the amplified products were resolved on 2.5% agarose gel electrophoresis and imaged using the Bio-Rad GelDoc documentation system.

### 4.4. In Vitro RNA Synthesis and Purification

The primer designing tool supported by NCBI was used to design primers targeting the sequences specific for N-gene, ORF1ab-gene, E-gene, and S-gene for in vitro transcription of mRNA. The 5′ end of the forward primer contains the T7 promoter sequence as well as the SARS-CoV-2 target sequence for in vitro synthesis of positive-strand mRNA. Supplementary Table S2 shows the primer sequences used for in vitro mRNA synthesis (forward and reverse). The viral RNA of the selected SARS-CoV-2 DNA fragment was synthesized in vitro using the MEGAscript^®^ in vitro transcription kit (# AM1334, Thermo Fisher Scientific, Waltham, MA, USA). The in vitro transcription was carried out in a 20 µL of a reaction containing 10× reaction buffer, enzyme mixture, dNTPs solution, and template DNA (500–1000 ng) of SARS-CoV-2 was followed by incubation of 16 h at 37 °C, and the addition of 1 µL of TURBO DNase at 37 °C for 15 min. The transcribed viral mRNA was further extracted and purified using the MEGAclear™ kit (#AM1908, Thermo Fisher Scientific, Waltham, MA, USA) and finally resuspended in TE buffer. The purity of the RNA was evaluated and quantified by the Bio-Rad gel documentation system (Supplementary Table S3). The purified viral mRNA was further used as a positive control template for the establishment of RT-LAMP assay.

### 4.5. RT-LAMP Assay Using In Vitro Transcribed SARS-CoV-2 Viral mRNA

A singleplex isothermal amplification for each target region using selected primer sets from the LAMP assay targeting N-gene (N-9), ORF1ab-gene (ORF1ab-2), E-gene (E-1) and S-gene (S-4) was carried out in the thermal cycler at a constant temperature of 63 °C for 1 h. The one-step RT-LAMP reaction mixture (25 µL) was prepared with 10× Isothermal buffer, 6 mM MgSO_4_, 1.4 mM dNTPs, 800 mM Betaine, 8 U Bst 2.0 WarmStart DNA polymerase, 5 U Reverse transcriptase (#M0380, New England Biolab, Ipswich, MA, USA), 1.6 µM FIP and BIP, 0.8 µM LF and LB, 0.2 µM F3 and B3, 1 µL of in vitro transcribed viral mRNA template and Nuclease free water (q.s.). In vitro transcribed RNA was used as a positive control template at a concentration of 10^6^ agµL^−1^, 10^5^ agµL^−1^, 10^4^ agµL^−1^, 10^3^ agµL^−1^, 10^2^ agµL^−1^, 10 agµL^−1^ and 1 agµL^−1^ in the RT-LAMP assay. The absorbance of the amplified products was observed for 1 h at 650 nm at every 1 min interval. Absorbance bar graphs were plotted for the maximum OD attained after 60 min for each dilution of viral RNA and a no-template control. Amplified products were visualized under visible light after the addition of 1 µL of 1000X SYBR Green I dye, and fluorescence was recorded. Fluorescence bar graphs were plotted for the fluorescence attained after 60 min of amplification of no template control (NTC) and viral RNA dilutions. Further same amplified products were resolved with the help of 2.5% agarose gel electrophoresis, and amplicons were captured with the help of the BioRad Gel-Doc documentation system.

### 4.6. RT-LAMP Using FAM and Biotin Tagged Primers

As described above, four primer sets were finally selected to perform the RT-LAMP with6-FAM and biotin-tagged primer sets. The same reaction components were used for the amplification of SARS-CoV-2 RNA under isothermal conditions with 5′ 6-FAM and biotin-tagged primers sets (N-9, ORF1ab-2, E-1, and S-4) (Supplementary Table S4). The RT-LAMP reaction for the N-gene and E-gene was performed using 1.6 µM of inner primers (FIP and BIP), 0.8 µM each of labeled and unlabeled loop primers (LF and LB), and 0.2 µM of outer primers (F3 and B3). The reaction for the ORF1ab-gene and S-gene was performed using 1.6 µM each of labeled and unlabeled inner primers, 0.8 µM of loop primers, and 0.2 µM of outer primers. Amplification was observed with the help of absorbance and fluorescence through SYBR Green I dye and agarose gel electrophoresis.

### 4.7. LFD Detection of RT-LAMP Amplified In Vitro Transcribed Viral mRNA

Milenia HybriDetect dipsticks (#MGHD1) were used to visualize 6-FAM and biotin-labeled RT-LAMP amplified products. On the conjugation pad, 6-FAM and biotin-labeled RT-LAMP products conjugate with anti-FAM-labelled gold nanoparticles. This gold nanoparticle amplified product conjugates and diffuses across the nitrocellulose membrane via capillary action, interacting with the control and test lines to produce a red line. Amplified RT-LAMP products were diluted in running buffer at a ratio of 1:100 and allowed to interact with the strips for 5 min. The presence of the test line in conjunction with the control line indicated a positive sample for SARS-CoV-2, whereas the absence of the control line indicated a negative sample for SARS-CoV-2.

### 4.8. Preparation of Gold Nanoparticles Conjugates

Gold nanoparticles (GNPs) were conjugated with monoclonal anti-fluorescein (FITC) IgG antibody produced in mice (Sigma-Aldrich) using the Abcam Gold conjugation kit (40 nm, 20 OD). The kit was supplied with the antibody diluent, the lyophilized vial of gold 40 nm, the reaction buffer, and the quencher. All the components of the kit reagents were thawed to room temperature, and the stock of anti-FITC (2 mg mL^−1^) was diluted to 0.1 mg mL^−1^. The diluted antibody (12 µL) was added to the reaction buffer (42 µL), and the mixture (45 µL) was transferred to a vial of Abcam lyophilized gold nanoparticles followed by the incubation of 15 min at room temperature. Gold quencher (5 µL) was then added to the conjugated gold nanoparticles and allowed to incubate for 5 min at room temperature. Finally, the gold nanoparticles conjugated with anti-FITC (50 µL) having 20 OD were prepared with a red color appearance which can be visualized through the naked eye and stored at 4 °C till further use in the development of a lateral flow device.

### 4.9. Preparation of Self-Assembled Nucleic Acid-Based Lateral Flow Device

Sample pad (Type GFB-R7L), conjugate release pad (Type PT-R7), nitrocellulose membrane with an adhesive plastic backing of 250 µm thickness (Type CNPF-SN12-L2-P25), and absorbent pad (Type AP110) for the assembly of lateral flow device were procured from the Advanced Microdevices (Ambala Cantt, India). The lateral flow device (6.0 × 0.3 cm^2^) was assembled using 1.5 cm of a sample pad, 0.5 cm of conjugation release pad, 2.5 cm of nitrocellulose membrane, and 1.5 cm of the absorbent pad with 0.1 cm of overlapping between components. Streptavidin (1 mg mL^−1^) (Promega, Madison, WI, USA) and anti-mouse IgG (whole molecule)-rabbit (2 mg mL^−1^) (Sigma, St. Louis, MO, USA) were spotted at 0.5 cm on nitrocellulose membrane apart using 1.0 µL of volume onto the test and control band respectively. The spotted membrane was then allowed to dry at room temperature for 45 min.

### 4.10. Detection of RT-LAMP Amplified Products on Lateral Flow Device

The double run method [45] was used for the detection of RT-LAMP products in which instead of impregnation of conjugation release pad in ≥5 µL conjugated gold nanoparticles, 1.0 µL of the conjugated gold nanoparticle was pipetted on the conjugation release pad assembled in the lateral flow strip. The amplified product was diluted in the 1.5 µL microcentrifuge tube containing sample volume 5.0 µL and filter sterilized running buffer TBST 95.0 µL (20 mM Tris HCl, 150 mM NaCl, 0.05% Tween 20; pH 7.4). Once the running buffer flow to the absorbent pad, the pipette again 1.0 µL of conjugated gold nanoparticle on the conjugation release pad to increase the visibility of the test and control spots on the strip. Incubate the strips for 10 min. The experiment was performed with the RT-LAMP amplified negative template control and positive control mRNA (10 ng) of customized N-gene in triplicate to optimize the conditions for the lateral flow device (LFD) development. For the development of a lateral flow device, the assembled strip containing conjugated gold nanoparticles on the conjugation release pad was assembled in a cassette. In total, 20 µL of the diluted amplified sample, as mentioned earlier, was used on the sample application area. Later, the specificity of the LFD was assessed with RT-LAMP assay amplified mRNA of COVID-19 suspected samples targeting N-gene having Ct values 38.539 and 20.149 as provided by the real-time PCR assay. The presence of two red spots on the test and control area indicates the presence of SARS-CoV-2 mRNA in the sample, while the absence of a spot on the test area indicates a negative sample.

### 4.11. Validation of Lateral Flow Device Using SARS-CoV-2 Suspected Clinical RNA Samples

The human nasopharyngeal and oropharyngeal swabs samples were collected with the approval of the government for COVID-19 diagnostics as part of a routine procedure following the Indian Council of medical research (ICMR) guidelines. Approval was then taken from the Institutional Human Ethics Committee (IHEC) for the use of the remaining samples for COVID-19 diagnostics and the work mentioned in the current manuscript (Institutional Human Ethics Committee clearance certificate number: CSIR/IITR/IHEC/JUL/2020/1 Dated 28 July 2020). The samples were de-identified and anonymized by giving them unique code numbers and maintaining complete confidentiality. The IHEC had approved the waiver of informed consent for this work considering the above situation and following ICMR guidelines (waiver of informed consent under clause No. 5.6 (category mentioned in Box-5.2) page number 53–54 of ICMR National Ethical Guidelines for Biomedical and Health Research involving human participants, 2017).

The human nasal and pharyngeal swab samples were analyzed to confirm the presence of SARS-CoV-2 by real-time PCR method. All the samples were rapidly processed for magnetic bead-based RNA isolation as per the manufacturer’s protocol (#A42352 MagMAX™ Viral/Pathogen Nucleic Acid Isolation Kit, Thermo Fisher Scientific, Waltham, MA, USA). A volume of 2 µL in 12.5 µL of reaction was used as an RNA template for SARS-CoV-2 detection using RT-LAMP. All the precautionary measures were taken while performing the whole study. The reverse transcriptase real-time monitoring was performed to determine the Ct values of the patient’s RNA samples using a real-time fluorescent RT-PCR kit for detecting SARS-CoV-2 (#G030010, BGI, Shenzhen, China). The tagged amplified products were visualized in 1:100 dilution on the Milenia HybriDetect dipsticks (#MGHD1). After completion of the study, the samples were discarded following the approved SOP No. CSIR-IITR/BS/002. Biosafety measures were taken for the disposal of waste generated in the testing facility.

### 4.12. Surface Characterization of Screen-Printed Gold Electrode

The topography images of the sample surface were measured with an NT-MDT NTEGRA Prima AFM in-situ inverted Olympus IX-71 microscope. The measurements were performed in tapping mode using a semi-contact scanning probe of the ETALON series (NT-MDT Spectrum Instruments, Tempe, AZ, USA). A topographical scan of the screen-printed gold electrode was obtained in mode height and variation in the current magnitude at a scale of 5 µm.

FTIR spectra of the unmodified and modified screen-printed Au were recorded on a Thermo Nicolet iS20 FTIR spectrometer with an attenuated total reflection (ATR) mode operation using Omnic™ 9.9.549 software. The spectral resolution was set at 4 cm^−1^ and measured from 4000 to 500 cm^−1^ wave number.

### 4.13. Development of Electrochemical Sensor for the SARS-CoV-2 Detection

Solutions for immobilization of both primer and spacer (β-ME) were dissolved in Tris-EDTA buffer solution, pH 7.4 (#93302 Sigma-Aldrich, Buchs, Switzerland) in the required concentration from stock concentration of 100 µM and 1 mM. Appropriate primer between F3 and FIP of the N-9 LAMP primer set was selected by immobilizing each primer (0.5 µM) and spacer (0.5 µM) on the separate gold electrode. The immobilization was undertaken overnight (~20 h) at 37 °C in the dark. Next, the appropriate concentration of primer to spacer required for the development of a Self-assembled Monolayer (SAM) on a gold surface was determined by varying their concentration from 0–0.5 µM. The complementary (N-gene specific RNA) and non-complementary (ORF1ab, E, and S-gene specific RNA) SARS-CoV-2 RNA of 1 ngµL^−1^ concentration was amplified by RT-LAMP and denatured by giving heat shock (95 °C for 10 min and rapid cooling at −20 °C) prior to hybridization. The denatured amplified product was diluted in hybridization buffer (SSPE; 2.98 M NaCl, 0.02 M EDTA, and 0.2 M phosphate buffer pH 7.4), and the optimum hybridization time between the mobilized primer-spacer and denatured amplified product was selected by varying the time from 1 to 4 h at 55 °C. The sensing performance was measured before and after hybridization with the template concentration ranging from 100 fgµL^−1^ to 1 ngµL^−1^.

### 4.14. Validation of Electrochemical Sensor Using SARS-CoV-2 Suspected Clinical RNA Samples

The electrochemical behavior was evaluated by recording the differential pulse voltammetry (DPV) and electrochemical impedance spectroscopy (EIS) against a one-electron redox system of Fe(CN)_6_^3−^/Fe(CN)_6_^4−^ using Multi Autolab/M204 controlled by Nova 2 software. The DPV scans were performed from −0.1 to 0.3 V (potential step: 25 mV;).EIS measurements were scanned from 10^3^ to 1 Hz, at 10 Hz/period and amplitude of 5 mV. The change in peak current (ΔI) and fractional coverage (Ψ_FC_) of the electrode were calculated as follows:

$$\Delta I=I_{\mathrm{Au}}-I_{H}$$

$$\Psi_{\mathrm{FC}}=1-\frac{R_{\mathrm{ct}}^{\mathrm{Au}}}{R_{\mathrm{ct}}^{H}}$$

where $R_{\mathrm{ct}}^{\mathrm{Au}}\;\mathrm{of}\;$bare gold electrode; ${\;R}_{\mathrm{ct}}^{H}\;\mathrm{of}\;\mathrm{hybridization}$.

## 5. Conclusions

For point-of-care settings, a simple amplification system with a multi-target approach and a variety of detection systems is highly adaptable. The multiple targets (N, ORF1ab, E, and S) of the SARS-CoV-2 genome, as well as rapid isothermal amplification and rapid visualization of the results on spectrophotometry, lateral-flow devices, and electrochemical sensors, are the strengths of the detection system. These multiple detection systems approach increases the specificity of the assay. The developed detection systems are sensitive and attained a notable sensitivity of 10 agµL^−1^ for N-gene, ORF1ab, and E-gene and 100 agµL^−1^ for S-gene with spectrophotometric and fluorometric detection systems. The lateral flow devices for all gene targets attained a sensitivity of 10 agµL^−1^. The electrochemical sensor attained a sensitivity of log 0.067 µA/pgµL^−1^/mm^2^ and LOD of log 1.79 ± 0.427 pgµL^−1^. This study compares various analytical methods for the detection of SARS-CoV-2. Spectrophotometric and lateral flow devices were found to be more sensitive than the electrochemical sensor in terms of detection limit and turn-around time. The study’s limitation is its inability to simultaneously identify all the gene targets in a single RT-LAMP assay. Future studies may focus on the development of multiplex RT-LAMP for the detection of SARS-CoV-2 viral mRNA, which makes the assay more adaptable in point-of-care settings. However, these quick, dependable, sensitive, and simple-to-use systems will provide a screen-and-go approach for not only SARS-CoV-2 and its emerging variants but also for other viral diseases.

## Figures and Tables

**Table 1:** Validation of LFD for clinical samples of SARS-CoV-2 viral mRNA.

Sample ID	Gender Female: F Male: M	Age	COVID-19 Wave	Ct Value	N-Gene	ORF1ab-Gene	E-Gene	S-Gene
S1	F	35	I	11.298	+	+	+	+
S2	M	32	I	12.293	+	+	+	+
S3	M	55	I	12.978	+	+	+	+
S4	M	45	II	16.205	+	+	+	_
S5	F	22	II	17.337	+	+	+	_
S6	M	19	I	17.984	+	+	+	+
S7	M	28	I	19.427	+	+	+	+
S8	F	47	II	19.915	+	+	+	_
S9	M	28	II	20.149	+	+	+	_
S10	F	12	II	20.612	+	+	+	_
S11	M	22	I	22.311	+	_	+	+
S12	M	38	I	23.486	+	_	_	+
S13	M	29	I	26.162	+	+	+	+
S14	M	20	I	27.535	+	+	+	+
S15	M	35	I	27.99	+	+	+	+
S16	F	23	I	38.539	_	_	_	_

**Table 2:** Validation of electrochemical sensor for clinical samples of SARS-CoV-2 viral mRNA.

Cyclic Threshold (Ct Value)	Average Current Change (A)	Results	Inference
16.205	−4.24 × 10^−6^	_	False negative
17.337	5.55 × 10^−6^	+	Positive
19.915	6.13 × 10^−6^	+	Positive
20.149	9.84 × 10^−5^	+	Positive
20.642	1.42 × 10^−6^	+	Positive
20.777	5.32 × 10^−5^	+	Positive
21.972	6.13 × 10^−5^	+	Positive
22.879	6.06 × 10^−5^	+	Positive
23.031	5.83 × 10^−5^	+	Positive
25.05	5.37 × 10^−5^	+	Positive
30.997	5.45 × 10^−5^	+	Positive
32.37	5.69 × 10^−5^	+	Positive
38.539	−8.3 × 10^−5^	_	Negative
40.815	−5.1 × 10^−7^	_	Negative
-	−3.04 × 10^−6^	_	Negative

## Data Availability

The data sets generated and/or analyzed during the study are available from the corresponding author upon reasonable request.

## Supplementary Materials

The following supporting information can be downloaded at: https://www.mdpi.com/article/10.3390/ijms232113105/s1.
