# Supplementary material for: Development and Clinical Validation of RT-LAMP-Based Lateral-Flow Devices and Electrochemical Sensor for Detecting Multigene Targets in SARS-CoV-2
Source: Int J Mol Sci. 2022 Oct 28;23(21):13105. doi: 10.3390/ijms232113105 (PMC9658514; doi:10.3390/ijms232113105)
Supplement: Supplementary file 1 [file ijms-23-13105-s001.zip › ijms-1970247-SI.pdf]

Supporting Information

Title: Development and clinical validation of RT-LAMP based lateral-flow devices and electrochemical sensor for detecting multi-gene targets in SARS-CoV-2

Apoorva Saxena[1], Pawankumar Rai[1], Srishti Mehrotra[1,2], Samiya Baby[3], Suman Singh[2,4], Vikas Srivastava[2,3], Smriti Priya[2,3] and Sandeep K. Sharma[1,2*]

Supplementary Tables

Table S1: Sequences of LAMP primers used in this study

Table S1a: Target Name- N-gene			
**Primer ID**	**Primers**	**Sequence (5′ – 3′)**	**PMID No.**
N-1	F3	TGGCTACTACCGAAGAGCT	32719001, 32635743
	B3	TGCAGCATTGTTAGCAGGAT	
	FIP	TCTGGCCCAGTTCCTAGGTAGTCCAGACGAATTCGTGGTGG	
	BIP	AGACGGCATCATATGGGTTGCACGGGTGCCAATGTGATCT	
	LF	GGACTGAGATCTTTCATTTTACCGT	
	LB	ACTGAGGGAGCCTTGAATACA	
N-2	F3	ACCAGGAACTAATCAGACAAG	32635743
	B3	GACTTGATCTTTGAAATTTGGATCT	
	FIP	TTCCGAAGAACGCTGAAGCGGAACTGATTACAAACATTGG CC	
	BIP	CGCATTGGCATGGAAGTCACAATTTGATGGCACCTGTGTA	
	LF	GGGGGCAAATTGTGCAATTTG	
	LB	CTTCGGGAACGTGGTTGACC	
N-3	F3	GCCAAAAGGCTTCTACGCA	32276051
	B3	TTGCTCTCAAGCTGGTTCAA	
	FIP	TCCCCTACTGCTGCCTGGAGGCAGTCAAGCCTCTTCTCG	
	BIP	TCTCCTGCTAGAATGGCTGGCATCTGTCAAGCAGCAGCAAA G	
	LF	TGGTGGACCCTCAGATTCAA	
	LB	ATGGCGGTGATGCTGCTCT	
N-4	F3	GTTGTTCGTTCTATGAAGACT	32547882
	B3	GACGTTGTTTTGATCGCG	
	FIP	CGAACGTCATGATACTCTAAAATGTCTGATAATGGACCCCA	
	BIP	CGAAATGCACCCCGCATTACCCACTGCGTTCTCCATTC	
	LF	TGTTCGTTTAGATGAAATC	
	LB	TGGTGGACCCTCAGATTCAA	
N-5	F3	CCAGAATGGAGAACGCAGTG	32626666
	B3	CCGTCACCACCACGAATT	
	FIP	AGCGGTGAACCAAGACGCAGGGCGCGATCAAAACAACG	
	BIP	AATTCCCTCGAGGACAAGGCGAGCTCTTCGGTAGTAGCCAA	
	LF	TTATTGGGTAAACCTTGGGGC	
	LB	TTCCAATTAACACCAATAGCAGTCC	

N-6	F3	CACAAGCTTTCGGCAGAC	32588005
	B3	CATCCAATTTGATGGCACC	
	FIP	CGGCCAATGTTTGTAATCAGTTCCTGGTCCAGAACAAACCCAA	
	BIP	CGTTCTTCGGAATGTCGCGCGTAGGTCAACCACGTTCC	
	LF	TTCCTGGTCCCCAAAATTTCC	
	LB	ATTGGCATGGAAGTCACACC	
N-7	F3	TGGACCCCAAAATCAGCG	32333644
	B3	GCCTTGTCCTCGAGGGAAT	
	FIP	CCACTGCGTTCTCCATTCTGGTAAATGCACCCCGCATTACG	
	BIP	CGCGATCAAAACAACGTCGGCCCTTGCCATGTTGAGTGAGA	
	LF	TGAATCTGAGGGTCCACCAAA	
	LB	GGTTTACCCAATAATACTGCGTCTT	
N-8	F3	AGATCACATTGGCACCCG	32333644
	B3	CCATTGCCAGCCATTCTAGC	
	FIP	TGCTCCCTTCTGCGTAGAAGCCAATGCTGCAATCGTGCTAC	
	BIP	GGCGGCAGTCAAGCCTCTTCCCTACTGCTGCCTGGAGTT	
	LF	GCAATGTTGTTCCTTGAGGAAGTT	
	LB	GTTCCTCATCACGTAGTCGCAACA	
N-9	F3	CTACCTAGGAACTGGGCC	32692666
	B3	AGAAGAGGCTTGACTGCC	
	FIP	GGTGTATTCAAGGCTCCCTCACCTATGGTGCTAACAAAGAC	
	BIP	AATCCTGCTAACAATGCTGCAATCCTGCTCCCTTCTGCGTAG	
	L F	GTTGCAACCCATATGATGC	
	L B	CTTCCTCAAGGAACAACAT	
N-10	F3	GCTGCAATCGTGCTACAACT	Self-designed
	B3	TTGCTCTCAAGCTGGTTCAA	
	FIP	TGCGACTACGTGATGAGGAACGTTGCCAAAAGGCTTCTACGC	
	BIP	AACTCCAGGCAGCAGTAGGGGAGCAGCAGCAAAGCAAGAG	
	L F	AGGCTTGACTGCCGCCTCT	
	L B	CTCCTGCTAGAATGGCTGGC	

Table S1b: Target Name- ORF-1ab-gene			
**Primer ID**	**Primer**	**Sequence (5′ – 3′)**	**Reference**
ORF-1AB-1	F3	TGCTTCAGTCAGCTGATG	32361529
	B3	TTAAATTGTCATCTTCGTCCTT	
	FIP	TCAGTACTAGTGCCTGTGCCCACAATCGTTTTTAAACGGGT	
	BIP	TCGTATACAGGGCTTTTGACATCTATCTTGGAAGCGACAACAA	
	LF	CTGCACTTACACCGCAA	
	LB	GTAGCTGGTTTTGCTAAATTCC	
ORF-1AB-2	F3	GGTATGATTTTGTAGAAAACCCA	32276116
	B3	CAACAGGAACTCCACTACC	
	FIP	GGCATCACAGAATTGTACTGTTTTTGCGTATACGCCAACTTAGG	
	BIP	AATGCTGGTATTGTTGGTGTACTGAGGTTTGTATGAAATCACCGAA	
	LF	AACAAAGCTTGGCGTACACGTTCA	
ORF-1AB-3	F3	GGATTTTGTGACTTAAAAGGTAAG	32692666
	B3	GCACTTACACCGCAAACC	
	FIP	CGGTACAGACTGTGTTTTTAAGTGTTGTACAAATACCTACAACTTGTG	
	BIP	TCTGCGGTATGTGGAAAGGTTATAAACGATTGTGCATCAGC	
	LF	AAACCCACAGGGTCATTAG	
	LB	GAACCCATGCTTCAGTC	
ORF-1AB-4	F3	TTGTGCTAATGACCCTGT	Self-designed
	B3	TCAAAAGCCCTGTATACGA	
	FIP	GATCACAACTACAGCCATAACCTTTTTACACTTAAAAACACAGTCTGT	
	BIP	GCTGATGCACAATCGTTTTTAAACGCATCAGTACTAGTGCCTGT	
	LF	CCACATACCGCAGACGGT	
	LB	GGTGTAAGTGCAGCCCGT	

Table S1c: Target Name- E-gene			
**Primer ID**	**Primers**	**Sequence (5′ – 3′)**	**PMID no.**
E-1	F3	TGAGTACGAACTTATGTACTCAT	32635743
	B3	TTCAGATTTTTAACACGAGAGT	
	FIP	ACCACGAAAGCAAGAAAAAGAAGTTCGTTTCGGAAGAGACAG	
	BIP	TTGCTAGTTACACTAGCCATCCTTAGGTTTTACAAGACTCACGT	
	LF	CGCTATTAACTATTAACG	
	LB	GCGCTTCGATTGTGTGCGT	

Table S1d: Target Name- S-gene			
**Primer ID**	**Primers**	**Sequence (5′ – 3′)**	**PMID No.**
S-1	F3	TCTATTGCCATACCCACAA	32276116
	B3	GGTGTTTTGTAAATTTGTTTGAC	
	FIP	CATTCAGTTGAATCACCACAAATGTGTGTTACCACAGAAATT CTACC	
	BIP	GTTGCAATATGGCAGTTTTTGTACATTGGGTGTTTTTGTCTTG TT	
	LF	ACTGATGTCTTGGTCATAGACACT	
	LB	TAAACCGTGCTTTAACTGGAATAGC	
S-2	F3	CTGACAAAGTTTTCAGATCCTCAG	32276051
	B3	AGTACCAAAAATCCAGCCTCTT	
	FIP	TCCCAGAGACATGTATAGCATGGAATCAACTCAGGACTTGTT CTTACC	
	BIP	TGGTACTAAGAGGTTTGATAACCCTGTTAGACTTCTCAGTGG AAGCA	
	LF	CCAAGTAACATTGGAAAAGAAA	
	LB	GTCCTACCATTTAATGATGGTGTTT	
S-3	F3	TCTTTCACACGTGGTGTT	32333644
	B3	GTACCAAAAATCCAGCCTC	
	FIP	CATGGAACCAAGTAACATTGGAAAACCTGACAAAGTTTTCAG ATCC	
	BIP	CTCTGGGACCAATGGTACTAAGAGGACTTCTCAGTGGAAGCA	
	LF	GAAAGGTAAGAACAAGTCCTGAGT	
	LB	CTGTCCTACCATTTAATGATGGTGT	
S-4	F3	GTGTTACCACAGAAATTCTACC	Self-designed
	B3	GTGCAAAAACTTCTTGGGT	
	FIP	GCATTCAGTTGAATCACCACAAATAGTGTCTATGACCAAGAC ATC	
	BIP	AGCAATCTTTTGTTGCAATATGGCTTTTTGTCTTGTTCAACAG CTAT	
	Loop B	CACAATTAAACCGTGCTTTAACTGG	

Table S1e: RT-LAMP primers selected for labelling			
Primer ID	Primers	Sequence (5′ – 3′)	PMID No.
N-5	F3	CCAGAATGGAGAACGCAGTG	32626666
	B3	CCGTCACCACCACGAATT	
	FIP	AGCGGTGAACCAAGACGCAGGGCGCGATCAAAACAACG	
	BIP	AATTCCCTCGAGGACAAGGCGAGCTCTTCGGTAGTAGCCAA	
	LF	TTATTGGGTAAACCTTGGGGC	
	LB	TTCCAATTAACACCAATAGCAGTCC	
	FIP*	Biotin AGCGGTGAACCAAGACGCAGGGCGCGATCAAAACAACG	
	BIP*	FAM AATTCCCTCGAGGACAAGGCGAGCTCTTCGGTAGTAGCCAA	
N-9	F3	CTACCTAGGAACTGGGCC	32692666
	B3	AGAAGAGGCTTGACTGCC	
	FIP	GGTGTATTCAAGGCTCCCTCACCTATGGTGCTAACAAAGAC	
	BIP	AATCCTGCTAACAATGCTGCAATCCTGCTCCCTTCTGCGTAG	
	LF	GTTGCAACCCATATGATGC	
	LB	CTTCCTCAAGGAACAACAT	
	LF*	Biotin GTTGCAACCCATATGATGC	
	LB*	FAM CTTCCTCAAGGAACAACAT	
ORF-1AB-1	F3	TGCTTCAGTCAGCTGATG	32361529
	B3	TTAAATTGTCATCTTCGTCCTT	
	FIP	TCAGTACTAGTGCCTGTGCCCACAATCGTTTTTAAACGGGT	
	BIP	TCGTATACAGGGCTTTTGACATCTATCTTGGAAGCGACAACAA	
	LF	CTGCACTTACACCGCAA	
	LB	GTAGCTGGTTTTGCTAAATTCC	
	LF*	Biotin CTGCACTTACACCGCAA	
	LB*	FAM GTAGCTGGTTTTGCTAAATTCC	
ORF-1AB-2	F3	GGTATGATTTTGTAGAAAACCCA	32276116
	B3	CAACAGGAACTCCACTACC	
	FIP	GGCATCACAGAATTGTACTGTTTTTGCGTATACGCCAACTTAGG	
	BIP	AATGCTGGTATTGTTGGTGTACTGAGGTTTGTATGAAATCACCGAA	
	LF	AACAAAGCTTGGCGTACACGTTCA	
	FIP*	Biotin GGCATCACAGAATTGTACTGTTTTTGCGTATACGCCAACTTAGG	
	BIP*	FAM AATGCTGGTATTGTTGGTGTACTGAGGTTTGTATGAAATCACCGAA	
E-1	F3	TGAGTACGAACTTATGTACTCAT	32635743
	B3	TTCAGATTTTTAACACGAGAGT	
	FIP	ACCACGAAAGCAAGAAAAAGAAGTTCGTTTCGGAAGAGACAG	

	BIP	TTGCTAGTTACACTAGCCATCCTTAGGTTTTACAAGACTCACGT	
	LF	CGCTATTAACTATTAACG	
	LB	GCGCTTCGATTGTGTGCGT	
	LF*	Biotin CGCTATTAACTATTAACG	
	LB*	FAM GCGCTTCGATTGTGTGCGT	
S-1	F3	TCTATTGCCATACCCACAA	32276116
	B3	GGTGTTTTGTAAATTTGTTTGAC	
	FIP	CATTCAGTTGAATCACCACAAATGTGTGTTACCACAGAAATTCTAC C	
	BIP	GTTGCAATATGGCAGTTTTTGTACATTGGGTGTTTTTGTCTTGTT	
	LF	ACTGATGTCTTGGTCATAGACACT	
	LB	TAAACCGTGCTTTAACTGGAATAGC	
	LF*	Biotin ACTGATGTCTTGGTCATAGACACT	
	LB*	FAM TAAACCGTGCTTTAACTGGAATAGC	
S-4	F3	GTGTTACCACAGAAATTCTACC	Self-designed
	B3	GTGCAAAAACTTCTTGGGT	
	FIP	GCATTCAGTTGAATCACCACAAATAGTGTCTATGACCAAGACATC	
	BIP	AGCAATCTTTTGTTGCAATATGGCTTTTTGTCTTGTTCAACAGCTAT	
	LF	CACAATTAAACCGTGCTTTAACTGG	
	FIP*	Biotin GCATTCAGTTGAATCACCACAAATAGTGTCTATGACCAAGACATC	
	BIP*	FAM AGCAATCTTTTGTTGCAATATGGCTTTTTGTCTTGTTCAACAGCTAT	

Table S2: Primers for invitro transcription of viral mRNA used in this study

Target	Forward primer (5'-3')	Reverse primer (5'-3')
N-gene	**TAATACGACTCACTATAGGG**GTTGTTCGTTCTATGAAGACT	GACTTGATCTTTGAAATTTGGATCT
ORF 1ab-gene	**TAATACGACTCACTATAGGG**GGATTTTGTGACTTAAAAGGTAAG	CAACAGGAACTCCACTACC
E-gene	**TAATACGACTCACTATAGGG**TGAGTACGAACTTATGTACTCAT	TTCAGATTTTTAACACGAGAGT
S-gene	**TAATACGACTCACTATAGGG**TCTATTGCCATACCCACAA	GGTGTTTTGTAAATTTGTTTGAC

*RNA polymerase Promoter-(T7 **TAATACGACTCACTATAGGG)**

Table S3: Yield and purity ratio of invitro transcribed viral mRNA used in the study

Target	Concentration (ng/µl)	A260/280	A260/230
N-gene	998.8	2.20	2.34
ORF1ab-gene	1246.6	2.27	2.43
E-gene	3022.9	2.14	2.40
S-gene	3398.5	2.15	2.29

Table S4: Primers selected for the establishment of RT-LAMP based LFD assay

Primer ID	Primers	Sequence (5′ – 3′)	PMID No.
**N-gene (N-9)**	F3	CTACCTAGGAACTGGGCC	32692666
	B3	AGAAGAGGCTTGACTGCC	
	FIP	GGTGTATTCAAGGCTCCCTCACCTATGGTGCTAACAAAGAC	
	BIP	AATCCTGCTAACAATGCTGCAATCCTGCTCCCTTCTGCGTAG	
	LF	GTTGCAACCCATATGATGC	
	LB	CTTCCTCAAGGAACAACAT	
	LF*	Biotin GTTGCAACCCATATGATGC	
	LB*	FAM CTTCCTCAAGGAACAACAT	
**ORF1ab-gene (ORF-1AB-2)**	F3	GGTATGATTTTGTAGAAAACCCA	32276116
	B3	CAACAGGAACTCCACTACC	
	FIP	GGCATCACAGAATTGTACTGTTTTTGCGTATACGCCAACTTAGG	
	BIP	AATGCTGGTATTGTTGGTGTACTGAGGTTTGTATGAAATCACCGAA	
	LF	AACAAAGCTTGGCGTACACGTTCA	
	FIP*	Biotin GGCATCACAGAATTGTACTGTTTTTGCGTATACGCCAACTTAGG	
	BIP*	FAM AATGCTGGTATTGTTGGTGTACTGAGGTTTGTATGAAATCACCGAA	
**E-gene (E-1)**	F3	TGAGTACGAACTTATGTACTCAT	32635743
	B3	TTCAGATTTTTAACACGAGAGT	
	FIP	ACCACGAAAGCAAGAAAAAGAAGTTCGTTTCGGAAGAGACAG	
	BIP	TTGCTAGTTACACTAGCCATCCTTAGGTTTTACAAGACTCACGT	
	LF	CGCTATTAACTATTAACG	
	LB	GCGCTTCGATTGTGTGCGT	
	LF*	Biotin CGCTATTAACTATTAACG	
	LB*	FAM GCGCTTCGATTGTGTGCGT	
**S-gene (S-4)**	F3	GTGTTACCACAGAAATTCTACC	Self-designed
	B3	GTGCAAAAACTTCTTGGGT	
	FIP	GCATTCAGTTGAATCACCACAAATAGTGTCTATGACCAAGACATC	
	BIP	AGCAATCTTTTGTTGCAATATGGCTTTTTGTCTTGTTCAACAGCTAT	
	LB	CACAATTAAACCGTGCTTTAACTGG	
	FIP*	Biotin GCATTCAGTTGAATCACCACAAATAGTGTCTATGACCAAGACATC	
	BIP*	FAM AGCAATCTTTTGTTGCAATATGGCTTTTTGTCTTGTTCAACAGCTAT	

Supplementary Figures

Figure S1: Sequences for customized DNA synthesis

1. E GENE (26232-26441)

TGAGTACGAACTTATGTACTCATTCGTTTCGGAAGAGACAGGTACGTTAATAGTTAA
TAGCGTACTTCTTTTTCTTGCTTTCGTGGTATTCTTGCTAGTTACACTAGCCATCCTTA
CTGCGCTTCGATTGTGTGCGTACTGCTGCAATATTGTTAACGTGAGTCTTGTAAAACC
TTCTTTTTACGTTTACTCTCGTGTTAAAAATCTGAA

2. N-GENE (28195-29323)

GTTGTTCGTTCTATGAAGACTTTTTAGAGTATCATGACGTTCGTGTTGTTTTAGATTT
CATCTAAACGAACAAACTAAAATGTCTGATAATGGACCCCAAAATCAGCGAAATGC
ACCCCGCATTACGTTTGGTGGACCCTCAGATTCAACTGGCAGTAACCAGAATGGAGA
ACGCAGTGGGGCGCGATCAAAACAACGTCGGCCCCAAGGTTTACCCAATAATACTG
CGTCTTGGTTCACCGCTCTCACTCAACATGGCAAGGAAGACCTTAAATTCCCTCGAG
GACAAGGCGTTCCAATTAACACCAATAGCAGTCCAGATGACCAAATTGGCTACTAC
CGAAGAGCTACCAGACGAATTCGTGGTGGTGACGGTAAAATGAAAGATCTCAGTCC
AAGATGGTATTTCTACTACCTAGGAACTGGGCCAGAAGCTGGACTTCCCTATGGTGC
TAACAAAGACGGCATCATATGGGTTGCAACTGAGGGAGCCTTGAATACACCAAAAG
ATCACATTGGCACCCGCAATCCTGCTAACAATGCTGCAATCGTGCTACAACTTCCTC
AAGGAACAACATTGCCAAAAGGCTTCTACGCAGAAGGGAGCAGAGGCGGCAGTCA
AGCCTCTTCTCGTTCCTCATCACGTAGTCGCAACAGTTCAAGAAATTCAACTCCAGG
CAGCAGTAGGGGAACTTCTCCTGCTAGAATGGCTGGCAATGGCGGTGATGCTGCTCT
TGCTTTGCTGCTGCTTGACAGATTGAACCAGCTTGAGAGCAAAATGTCTGGTAAAGG
CCAACAACAACAAGGCCAAACTGTCACTAAGAAATCTGCTGCTGAGGCTTCTAAGA
AGCCTCGGCAAAAACGTACTGCCACTAAAGCATACAATGTAACACAAGCTTTCGGC
AGACGTGGTCCAGAACAAACCCAAGGAAATTTTGGGGACCAGGAACTAATCAGACA
AGGAACTGATTACAAACATTGGCCGCAAATTGCACAATTTGCCCCCAGCGCTTCAGC
GTTCTTCGGAATGTCGCGCATTGGCATGGAAGTCACACCTTCGGGAACGTGGTTGAC
CTACACAGGTGCCATCAAATTGGATGACAAAGATCCAAATTTCAAAGATCAAGTC

3. S-GENE (23693-23937)

TCTATTGCCATACCCACAAATTTTACTATTAGTGTTACCACAGAAATTCTACCAGTGT
CTATGACCAAGACATCAGTAGATTGTACAATGTACATTTGTGGTGATTCAACTGAAT
GCAGCAATCTTTTGTTGCAATATGGCAGTTTTTGTACACAATTAAACCGTGCTTTAAC
TGGAATAGCTGTTGAACAAGACAAAAACACCCAAGAAGTTTTTGCACAAGTCAAAC
AAATTTACAAAACACC

4.ORF1ab-GENE (13286-14140)

GGATTTTGTGACTTAAAAGGTAAGTATGTACAAATACCTACAACTTGTGCTAATGAC
CCTGTGGGTTTTACACTTAAAAACACAGTCTGTACCGTCTGCGGTATGTGGAAAGGT
TATGGCTGTAGTTGTGATCAACTCCGCGAACCCATGCTTCAGTCAGCTGATGCACAA
TCGTTTTTAAACGGGTTTGCGGTGTAAGTGCAGCCCGTCTTACACCGTGCGGCACAG
GCACTAGTACTGATGTCGTATACAGGGCTTTTGACATCTACAATGATAAAGTAGCTG
GTTTTGCTAAATTCCTAAAAACTAATTGTTGTCGCTTCCAAGAAAAGGACGAAGATG
ACAATTTAATTGATTCTTACTTTGTAGTTAAGAGACACACTTTCTCTAACTACCAACA
TGAAGAAACAATTTATAATTTACTTAAGGATTGTCCAGCTGTTGCTAAACATGACTT
CTTTAAGTTTAGAATAGACGGTGACATGGTACCACATATATCACGTCAACGTCTTAC
TAAATACACAATGGCAGACCTCGTCTATGCTTTAAGGCATTTTGATGAAGGTAATTG
TGACACATTAAAAGAAATACTTGTCACATACAATTGTTGTGATGATGATTATTTCAA
TAAAAAGGACTGGTATGATTTTGTAGAAAACCCAGATATATTACGCGTATACGCCAA
CTTAGGTGAACGTGTACGCCAAGCTTTGTTAAAAACAGTACAATTCTGTGATGCCAT
GCGAAATGCTGGTATTGTTGGTGTACTGACATTAGATAATCAAGATCTCAATGGTAA
CTGGTATGATTTCGGTGATTTCATACAAACCACGCCAGGTAGTGGAGTTCCTGTTG

***Reference genome:** Severe acute respiratory syndrome coronavirus 2 isolate Wuhan-Hu-1, complete genome NCBI Reference Sequence: NC_045512.2

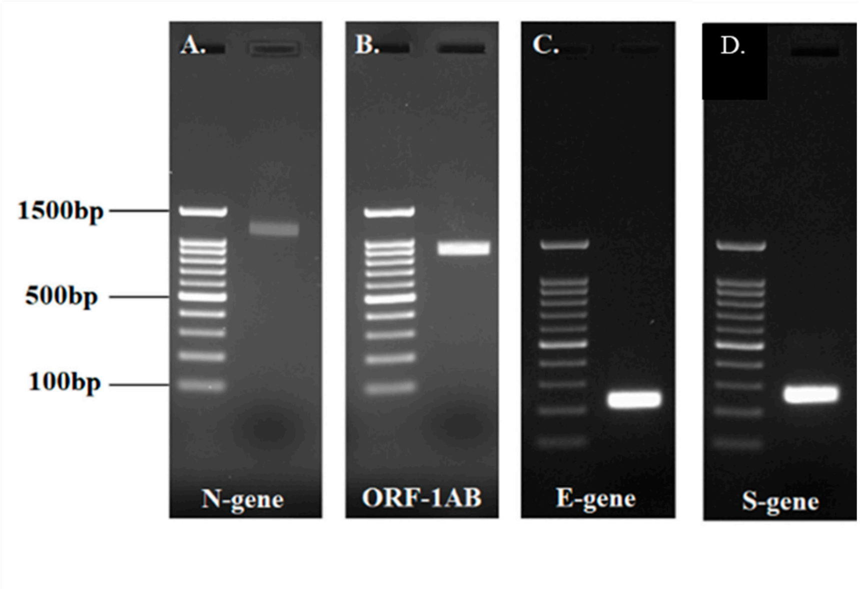

Figure S2: PCR amplification and purification of customized DNA sequence of SARS-CoV-2. PCR amplified and purified product visualised on agarose gel of **A)** N-gene (1129bp), **B)** ORF1ab-gene (855bp), **C)** E-gene (210bp),and, **D)** S-gene (245bp). L- DNA ladder (#MAGSPIN-21, APSLABS). All the experiments were replicated 3 times.

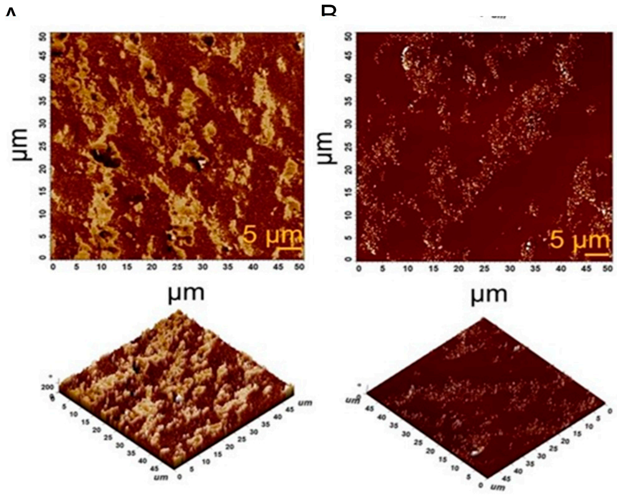

Figure S3: Surface characterization of screen-printed gold electrode through AFM. Topographical scan showing variation in the current magnitude at a scale of 5µm; A. screen-printed Au electrode (unmodified) and B. modified screen printed Au electrode/β-ME/ssDNA probe.

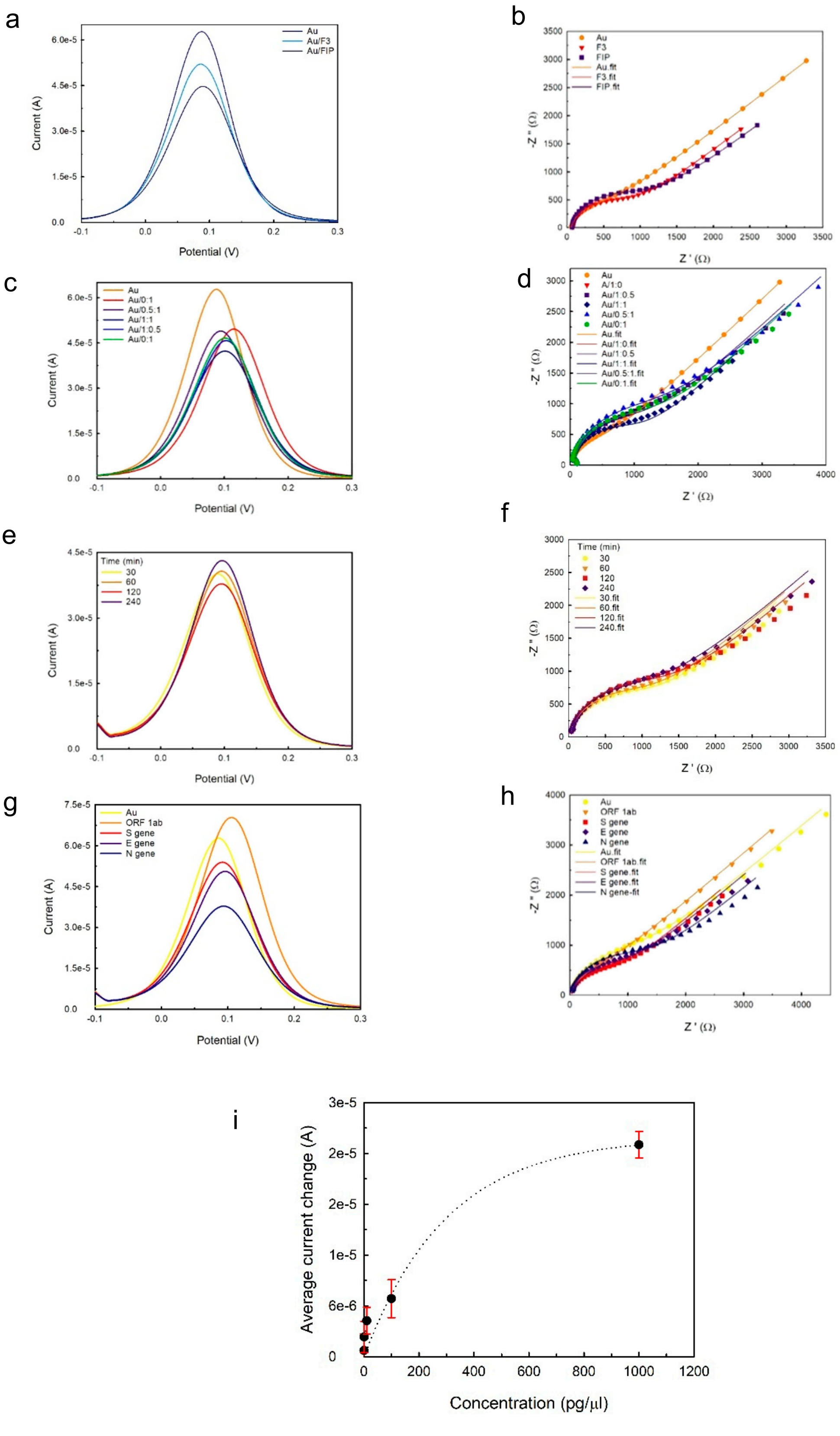

a
Au
Au/F3
Au/FIP
Current (A)
Potential (V)
b
Au
F3
FIP
Au.fit
F3.fit
FIP.fit
-Z " (Ω)
Z ' (Ω)
c
Au
Au/0:1
Au/0.5:1
Au/1:1
Au/1:0.5
Au/0:1
Current (A)
Potential (V)
d
Au
A/1:0
Au/1:0.5
Au/1:1
Au/0.5:1
Au/0:1
Au.fit
Au/1:0.fit
Au/1:0.5
Au/1:1.fit
Au/0.5:1.fit
Au/0:1.fit
-Z " (Ω)
Z ' (Ω)
e
Time (min)
30
60
120
240
Current (A)
Potential (V)
f
Time (min)
30
60
120
240
30.fit
60.fit
120.fit
240.fit
-Z " (Ω)
Z ' (Ω)
g
Au
ORF 1ab
S gene
E gene
N gene
Current (A)
Potential (V)
h
Au
ORF 1ab
S gene
E gene
N gene
Au.fit
ORF 1ab.fit
S gene.fit
E gene.fit
N gene-fit
-Z " (Ω)
Z ' (Ω)
i
Average current change (A)
Concentration (pg/μl)

Figure S4: Differential pulse voltammogram and nyquist plot to determine the (a,b) primer type between F3 and FIP, (c,d) assembly of the spacer and primer, (e,f) effect of hybridisation time and (g,h) the response of non-complementary DNA (NcDNA) namely, ORF 1ab, S gene and E gene, (i) Standard plot between the average current change and concentration of customized RNA standards of N-gene
